# Modulation of host cell pathways by *Coxiella burnetii* Dot/Icm effectors

**DOI:** 10.1002/mlf2.70058

**Published:** 2025-12-25

**Authors:** Jingya Yuan, Yong Zhang, Lei Song, Zhao‐Qing Luo

**Affiliations:** ^1^ Department of Respiratory Medicine, Center of Infectious Diseases and Pathogen Biology, Key Laboratory of Organ Regeneration and Transplantation of The Ministry of Education, State Key Laboratory for Diagnosis and Treatment of Severe Zoonotic Infectious Diseases The First Hospital of Jilin University Changchun China

**Keywords:** bacterial virulence, intravacuolar pathogens, lysosome, Q fever, type IV secretion

## Abstract

*Coxiella burnetii*, the etiological agent of Q fever, is a significant intracellular bacterial pathogen. *C. burnetii* is a highly infectious pathogen that primarily targets pulmonary alveolar macrophages during natural infection. It can then disseminate to macrophages in other tissues and organs, leading to chronic infections. *C. burnetii* is capable of infecting a variety of cultured cells, including primary macrophages, macrophage‐like cells, epithelial cells, and fibroblasts. The virulence of *C. burnetii* is entirely dependent on the Dot/Icm type IVB secretion system (T4BSS), which delivers effectors into infected cells to modulate cellular pathways for the biogenesis of the Coxiella‐containing vacuole that supports its intracellular replication. A deeper understanding of how *C*. *burnetii* exploits host cell processes is essential for developing novel therapeutic strategies to combat infections caused by this important pathogen. This review summarizes the historical milestones and recent advances in our understanding of the structure and function of the *C. burnetii* Dot/Icm system and its effectors.

## INTRODUCTION

*Coxiella burnetii*, a Gram‐negative obligate intracellular bacterium with pleomorphic morphology ranging from spherical to rod‐shaped, is the causative agent of the globally occurring zoonotic disease Q fever^1^. Humans become infected primarily through inhalation of contaminated dust aerosols or contact with excretions (such as milk, urine, and feces) and birth products of infected ruminants^2^. In humans, Q fever may be asymptomatic or manifest as a mild, self‐limiting influenza‐like illness. However, in some case, acute Q fever can progress to more severe conditions, including interstitial pneumonia or hepatitis^3^. While acute Q fever is treatable, approximately 1%–5% of patients may develop chronic Q fever (CQ) years after the initial infection^4^. The primary clinical manifestation of CQ is endocarditis, although chronic hepatitis, osteoarticular diseases, and chronic pulmonary diseases are less commonly observed^1^. Treatment for CQ generally involves a prolonged combination of doxycycline and hydroxychloroquine for 18–24 months. However, this prolonged duration of treatment and the associated severe side effects pose significant challenges to patient compliance and effective disease management^5^. Additionally, *C*. *burnetii* is highly infectious, with as few as 10 bacteria capable of causing disease^6^, ^7^. Its ability to survive in harsh environments and spread through contaminated aerosols prompted the categorization of this bacterium as a potential bioterrorism agent^8^. Consequently, a comprehensive understanding of the pathogen and its mechanisms of pathogenesis is essential for the development of more effective and novel therapeutic strategies.

**Figure 1:**
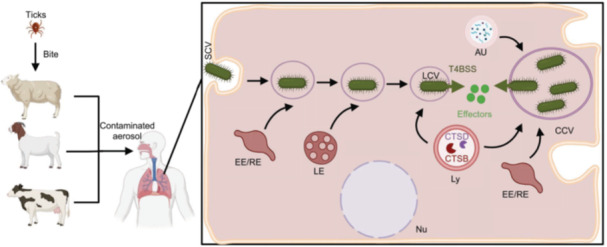
Schematic of *Coxiella burnetii* infection and its intracellular lifestyle. In the natural environment, ticks serve as the primary reservoir and vector of *C. burnetii*, transmitting the bacterium to livestock through their bites. The main sources of human infection are cattle, sheep, and goats, with humans typically contracting the bacterium by inhaling contaminated aerosols from the environment. Upon entering host cells, *C. burnetii* resides in a phagosome, which sequentially fuses with EE, LE, and lysosomes, ultimately forming a CCV that exhibits characteristics of a phagolysosome. Following acidification of the CCV, bacterial metabolism is activated, triggering the synthesis and assembly of functional T4BSS. This enables *C. burnetii* to translocate a range of effectors into the host cell through the activated T4BSS, facilitating the expansion and maturation of the CCV. This process enables the bacterium to complete its replication cycle within the vacuole. Au, autophagosome; CCV, *Coxiella*‐containing vacuole; CTSB, cathepsin B; CTSD, cathepsin D; EE, early endosome; LE, late endosome; Ly, lysosome; Nu, nucleus; RE, recycling endosome; T4BSS, type IVB secretion system. Created in BioRender. Song, L. (2025) https://BioRender.com/rspjc76.

*C*. *burnetii* exhibits a biphasic life cycle, alternating between the small cell variant (SCV) and large cell variant (LCV) forms^9^. The SCV is metabolically inactive and highly resistant to environmental challenges, serving as the extracellular survival form of the bacterium^1^. Upon infection, SCVs differentiate into metabolically active LCVs within host cells (Figure 1), allowing the bacterium to complete its growth cycle^10^. Lipopolysaccharide (LPS) is a critical virulence determinant for *C*. *burnetii*, modulating host immune responses. Based on the structure of LPS on the bacterial surface, *C*. *burnetii* exists in two distinct phase forms: the virulent Nine Mile phase I (NMI) strain and the less virulent Nine Mile phase II (NMII) strain^11^. The LPS of the NMI strain is complete, containing an O‐antigen with unusual sugars^12^. In contrast, the LPS of the NMII strain is truncated and lacks the O‐antigen polysaccharide chain, resulting in a less virulent strain that can be safely handled in biosafety Level 2 (BSL2) laboratory^13^. This phase variation may arise from spontaneous deletions of multiple genes on the chromosome^14^. Although the lipid A structures of these two strains are similar to each other, they differ substantially from the standard lipid A found in intestinal bacteria. Studies have shown that NMII strains are more efficiently internalized by macrophages and non‐phagocytic cells compared to NMI strains^15^, ^16^. In cultured human macrophages, both strains exhibit comparable proliferation rates^17^. Due to their lower virulence and the ability to be safely handled, NMII strains are widely used for studying host‐pathogen interactions under normal containment conditions.

**Figure 2:**
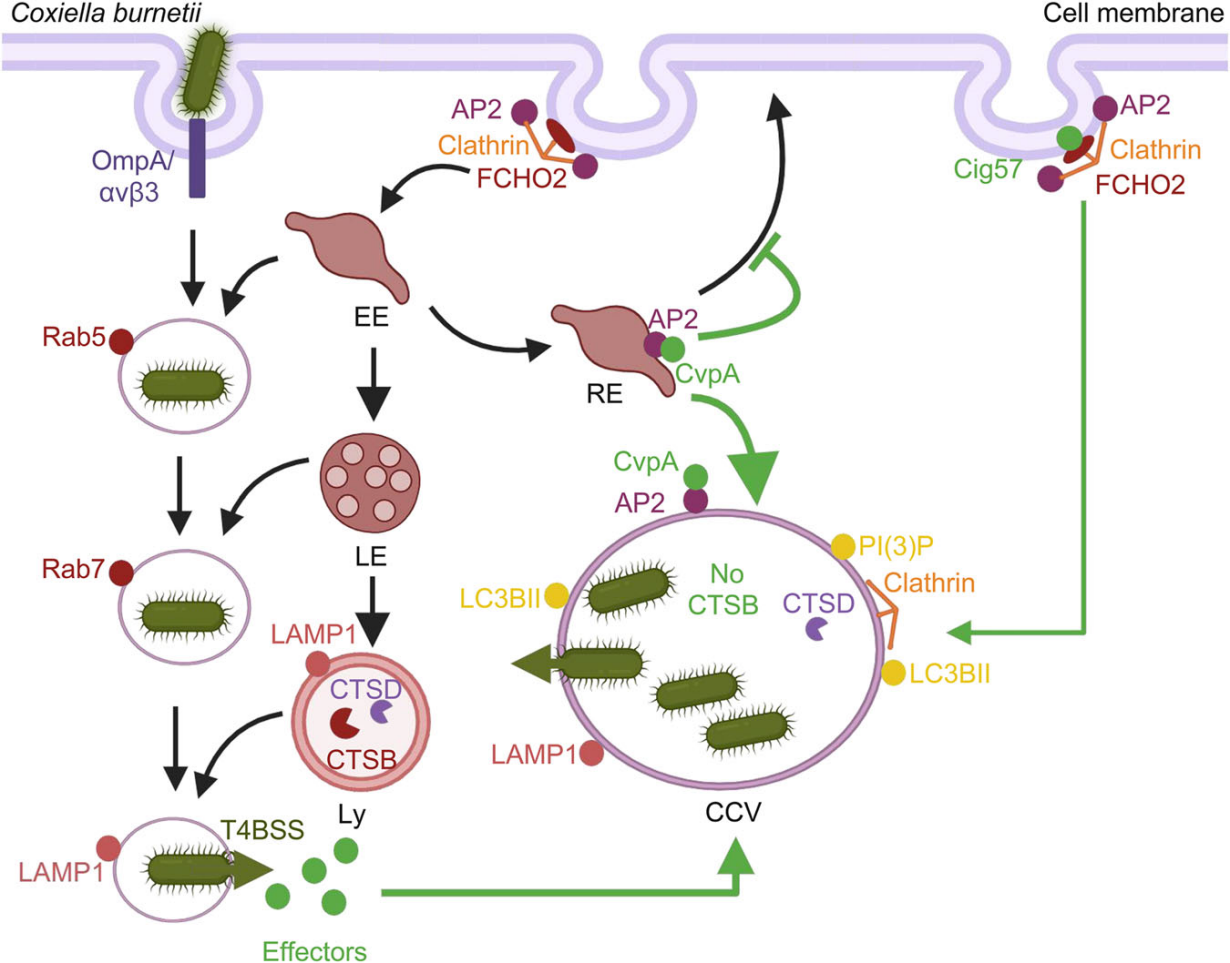
Modulation of host clathrin‐mediated vesicle trafficking by *C. burnetii* Dot/Icm effectors. After being internalized, *C*. *burnetii* resides in an endocytic vacuole decorated with Rab5, which facilitates its fusion with EEs. Subsequently, Rab7 is recruited to the vacuole, promoting its fusion with LEs and eventually directing the vacuole toward the lysosome network. Upon fusion with lysosomes, the vacuole becomes acidified, activating the Dot/Icm system T4BSS. CvpA binds to AP2 on RE, while Cig57 interacts with FCHO2 at the plasma membrane or other compartments in the cell, disrupting clathrin‐mediated vesicle trafficking. This interaction aids in the recruitment of clathrin to CCVs, facilitating their formation and maturation. Created in BioRender. Song, L. (2025) https://BioRender.com/7363j3q.

During natural infections, *C. burnetii* primarily infects monocytes and macrophages. However, there have also been reports of its ability to infect non‐phagocytic cells, including epithelial and endothelial cells. Numerous studies have demonstrated that the bacterium is capable of infecting a broad range of cell types in vitro infection models^18^, ^19^. Phagocytes internalize the bacterium through classical phagocytosis, mediated by αvβ3 integrin and the complement receptor 3 (CR3)^16^. Nonphagocytic cells, on the other hand, require the outer membrane protein OmpA for bacterial uptake^20^ (Figure 2). Upon phagocytosis, the phagosome containing *C. burnetii* gradually matures into an acidic compartment with distinct lysosomal characteristics. Lysosomal markers, such as vacuolar proton ATPase, acid phosphatase, 5′‐nucleotidase, cathepsin D, and LAMPs 1, 2, and 3, are associated with this compartment^21^. The acidification of the phagosome activates the metabolism of SCVs, facilitating their conversion into metabolically active LCVs. This transition is accompanied by the upregulation of genes for Dot/Icm structure and effectors, which are then delivered into host cells^22^ (Figure 1). Recent studies employing cryo‐electron tomography have revealed that the Dot/Icm apparatus is detected in LCVs but not in SCVs^23^. This finding suggests that the expression and assembly of Dot/Icm type IVB secretion system (T4BSS) are tightly regulated during the biphasic transition.

## GENETICS OF *C*. *burnetii* AND IDENTIFICATION OF ITS VIRULENCE EFFECTORS

T4SSs are highly heterogeneous and structurally diverse nanomachines found in many bacterial species. These systems facilitate the transfer of primarily protein and/or DNA between bacterial cells, and in some cases, into eukaryotic cells^24^. Certain bacteria utilize T4SSs to transfer DNA or proteins to the extracellular environment or introduce DNA into cells without direct contact with the target cells^24^. T4SSs are classified into two subgroups: those similar to the VirB system of *Agrobacterium tumefaciens* and the Tra system of plasmid pKM101, which are referred to as IVA, and those resembling the Dot/Icm system of *Legionella pneumophila* and the Tra/Trb system of IncI plasmids (e.g., R64), known as IVB^25^.

The Dot/Icm secretion system was originally discovered in *L. pneumophila* in the late 1990s. Genetic analyses of this bacterium identified approximately 30 genes essential for intracellular replication and/or cytotoxicity toward mammalian cells^26^, ^27^, ^28^, ^29^, ^30^, ^31^, ^32^, ^33^, ^34^. These genes were designated as *dot* or *icm* by the research groups of Ralph Isberg and Howard Shuman, respectively. Notably, these genes also support plasmid conjugation between *L*. *pneumophila* and *E. coli* ^35^, ^36^, ^37^. In 2000, it was found that genes involved in the conjugation of IncI plasmids shared significant similarity with *dot*/*icm* genes of *L*. *pneumophila* ^38^, ^39^, further establishing that the *dot*/*icm* genes evolved from classic conjugative T4SS dedicated for DNA transfer. The first protein substrate of the *L. pneumophila* Dot/Icm system, known as RaIF, was reported in 2002^40^.

Genome analysis of *C*. *burnetii* revealed the presence of a gene set similar to the *L*. *pneumophila* Dot/Icm secretion system, suggesting a similar function^41^. Unlike *L*. *pneumophila*, where *dot*/*icm* genes are distributed in two separate loci (regions I and II), most *C*. *burnetii dot*/*icm* genes are concentrated within a contiguous 33‐kb DNA segment, with the *dotU* and *icmF* genes located elsewhere in the genome. *C*. *burnetii* encodes 23 out of the 26 Dot/Icm proteins present in *L*. *pneumophila*, but it lacks homologs for the chaperone protein IcmR and the inner membrane proteins DotJ and DotV^41^. The *dotB*, *icmS*, *icmW*, and *icmT* genes of *C. burnetii* can rescue corresponding mutants in *L. pneumophila*, whereas the *icmX*, *icmQ*, *dotM*, *dotL*, *dotN*, and *dotO* genes cannot, indicating pathogen‐specific interactions among Dot/Icm proteins. Notably, IcmQ of *C. burnetii* cannot compensate for the growth defect caused by the loss of its homologous gene in *L. pneumophila* ^35^, probably due to the fact that in *L. pneumophila*, IcmQ interacts with IcmR to prevent its polymerization. Importantly, the chaperone proteins IcmS and IcmW from *C. burnetii* can complement *L*. *pneumophila* mutants lacking the corresponding genes, suggesting a conserved mechanism for recognizing at least a subset of the Dot/Icm substrates between these two systems^42^. Transposon‐induced disruption of *dot/icm* genes abolished intracellular replication of *C. burnetii*, highlighting the essential role of T4BSS in its virulence^43^.

*C*. *burnetii* was first transformed with the shuttle plasmid pSKO(+)‐1000. This plasmid contains ars derived from the C. burnetii genome, which is cloned into a ColE1‐type replicon. This ColE1 replicon also encodes β‐lactamase, enabling successfully transformed *C. burnetii* to grow on media containing ampicillin, serving as a selection marker^44^, ^45^. Subsequently, the Heinzen lab successfully isolated a *C. burnetii* mutant and characterized it using the *Himar1* transposon mutagenesis, based on the marine spiral transposon family and the Vero cell system^46^. However, due to low transformation efficiency and the lack of suitable host‐free culture media, obtaining *C. burnetii* clones in a short time remains challenging, making genetic manipulation and phenotypic analysis difficult. A breakthrough was made in 2009 when the Heinzen lab developed the acidified cysteine‐citrate medium (ACCM), which enables axenic growth of *C*. *burnetiid* ^47^, ^48^. This advancement has significantly facilitated *C*. *burnetii* research, enabling the generation of subsequent mutants using the *Himar1* transposon and the development of RSF1010‐based shuttle vectors for gene expression in this bacterium^47^, ^49^, ^50^. Further efforts led to the establishment of a Tn7 system for a single‐copy, site‐specific cis complementation method^49^, ^51^, as well as an anhydrotetracycline‐based^49^ and isopropyl β‐d‐1‐thiogalactopyranoside inducible expression system^52^, ^53^.

Early success in the identification of *C*. *burnetii* type IV effectors (T4Es) was achieved by combining bioinformatics analysis with the use of *L*. *pneumophila* as a surrogate to determine Dot/Icm‐dependent protein translocation^50^, ^54^. Chen et al. constructed a genomic library of *C. burnetii* and employed bacterial two‐hybrid technology to screen for proteins that interact with DotF^50^, a protein previously shown to bind to at least a subset of Dot/Icm substrates in *L. pneumophila* ^55^. Using *L*. *pneumophila* as a surrogate along with the CyaA and TEM1 fusion reporters, they showed that six of these proteins were Dot/Icm substrates. Then, these scientists employed bioinformatic approaches to identify 42 proteins homologous to known Dot/Icm substrates from both *L. pneumophila* and *C. burnetii*, of which 26 were shown to be translocated by the *L. pneumophila* Dot/Icm system^50^. Carey et al. used a similar strategy to identify 7 *C. burnetii* T4Es^54^. By using the three bioinformatic screening criteria of PmrA‐responsive promoter elements^56^, E‐block motifs^57^, and homology to known effectors^58^, Weber et al. successfully identified 53 *C. burnetii* T4Es^59^.

The conservation of T4Es in *C. burnetii* is relatively low across different strains. Comparative genomic analysis revealed significant variability in effectors among different *C. burnetii* isolates, with only 12 effectors being conserved across all isolates^59^. Furthermore, many T4E genes harbor stop codons that cause the loss of the C‐terminal translocation signals^59^, preventing these substrates from being delivered into host cells during infection. These findings support the idea that different *C. burnetii* pathotypes may harbor distinct pools of effectors. Additionally, some effector genes have also undergone frameshift mutations, resulting in the formation of multiple pseudogenes. These genetic variations likely correlate with the diverse effector requirements associated with different disease states during infection^59^.

To investigate whether a single T4E gene is essential for intracellular replication and maturation of the Coxiella‐containing vacuole (CCV), Weber et al. employed the *Himar1* transposon to perform random mutagenesis, generating 20 distinct Dot/Icm effector mutants. These mutants were then used to infect J774A.1 and HeLa cells, revealing that six of the mutants failed to replicate within host cells. Importantly, these mutants did not exhibit detectable growth defects when cultured in ACCM‐2 medium, suggesting that the observed intracellular replication deficiency was not due to intrinsic growth defects. Instead, the inability to replicate within host cells was likely due to the absence of an effector crucial for establishing the intracellular niche permissive for bacterial growth^59^. Using transposon insertion sequencing technology (INSeq), Crabill et al. identified seven T4Es necessary for optimal biogenesis of the CCV from an arrayed mutant library^60^. These results reveal that loss of a single T4E gene often leads to severe defects in intracellular growth of *C. burnetii*, in sharp contrast to *L. pneumophila*, where mutants defective in a single T4E gene rarely exhibit detectable defects in intracellular replication^61^, ^62^. To date, 130 T4Es of *C. burnetii* have been reported, accounting for 6.092% of the coding sequences in the reference strain RSA493 (2134 coding sequences) (Table 1). Understanding the mechanism of action of T4Es remains a critical focus in the study of *C. burnetii* pathogenesis.

**Table 1:** Dot/Icm substrates of *Coxiella burnetii*.

Gene ID	Alias	Function	Size (bp)	References
CBUA0006	CpeA	None	1026	^63^
CBU0012	None	None	812	^64^
CBUA0013	CpeB	None	732	^63^
CBUA0014	CpeC	None	234	^63^
CBUA0015	CpeD	None	684	^63^
CBU0015	None	None	717	^54^
CBUA0016	CpeE	None	1026	^63^
CBU0021	CvpB	Promoting the accumulation of PI(3)P on CCVs	2430	^65^, ^66^
CBUA0023	CpeF	None	702	^63^
CBUA0028	ToxP	None	456	^52^
CBUA0027	AntitoxP	None	345	^52^
CBU0041	CirA	Stimulating the GTPase activity of RhoA	2133	^67^
CBU0062	None	None	1506	^68^
CBU0069	AnkP	None	2164	^64^
CBU0072	AnkA	None	1200	^64^
CBU0077	MceA	None	792	^69^
CBU0096	None	Predicted phospholipase D	1176	^70^
CBU0113	None	None	213	^64^
CBU0114	AnkB	None	483	^11^, ^20^
CBU0122	None	None	279	^71^
CBU0145	None	None	513	^43^
CBU0175	CstK	A key protein kinase, recruiting the host protein TBC1D5 to CCVs	741	^72^
CBU0183	None	None	273	^59^
CBU0201	AnkC	None	396	^70^
CBUD0212	None	None	717	^59^
CBU0270	None	None	1221	^59^
CBU0295	None	None	1470	^54^
CBU0307a	None	Interfering with apoptosis	246	^64^
CBU0329	None	None	777	^54^
CBU0344	None	None	535	^59^
CBU0372	None	None	1365	^59^
CBU0375	None	None	723	^59^
CBUD0376	None	None	3012	^59^
CBU0388	CetCb2	Modulation of the MAPK pathway	4179	^59^, ^68^
CBU0393	None	None	2097	^59^
CBU0410	None	None	1737	^59^
CBU0414	None	None	1236	^59^
CBU0425	CirB	Inhibiting the hydrolytic activity of the proteasome	1368	^73^
CBU0447	AnkF	Recruiting endogenous vimentin to CCV	555	^74^
CBU0469	None	None	204	^59^
CBU0485	None	None	1617	^64^
CBU0505	Cig14	None	618	^70^
CBU0513	CinF	Dephosphorylating IΚBα	1149	^75^
CBU0519	DedA	Encoding a SNARE‐like domain‐containing protein	609	^70^
CBU0542	LigA	Encoding a predicted DNA ligase	2022	^70^
CBU0547	None	None	1686	^70^
CBU0590	None	None	174	^59^
CBU0606	None	None	1389	^59^
CBU0626	CvpF	Recruiting Rab26 to CCVs and promoting the accumulation of LC3BII on CCVs	2088	^76^
CBU0635	None	None	1491	^64^
CBU0637	None	None	762	^64^
CBU0665	CvpA	Targeting the AP2‐clathrin‐mediated vesicle trafficking pathway to CCVs	987	^77^
CBU0773	None	None	471	^59^
CBU0781	AnkG	Regulating the expression of apoptosis‐related genes	1017	^64^
CBU0794	None	None	1395	^59^
CBU0801	Riml	None	438	^70^
CBU0814	None	None	1854	^59^
CBU0822	CbFic2	In the monomeric state, AMPylating host cell histone H3, while upon binding to DNA, deAMPylating on H3	1137	^78^
CBU0881	None	None	666	^59^
CBU0885	CetCb4	Modulation of the MAPK pathway	1167	^59^, ^68^
CBU0937	MceB	Locating within the mitochondria and homologous to bacterial porin proteins/porin residing in the outer membrane of *C. burnetii*	1452	^79^, ^80^
CBU0978	None	None	468	^68^
CBU1043	GacA.4	None	645	^64^
CBU1045a	None	None	114	^59^
CBU1063	None	None	1407	^68^
CBU1079	None	None	597	^59^
CBUD1102	None	None	576	^59^
CBUD1108	None	None	2223	^54^
CBUD1110	None	None	579	^59^
CBU1150	None	None	688	^59^
CBU1198	None	None	537	^59^
CBU1206	None	Encoding a predicted sterol reductase	1380	^70^
CBU1213	AnkI	None	1974	^70^
CBU1217	NopA	Interacting with the Ran GTPase	1494	^70^
CBUD1251	None	None	1002	^43^
CBU1314	None	Blocking the polymerase‐associated factor 1 (PAF1) complex	645	^81^
CBU1366	Cig40	Encoding a coiled‐coil domain‐containing protein	333	^70^
CBU1370	CbEPF1	Binding to the host VAP family proteins and mediating the formation of membrane contact sites between host lipid droplets and the endoplasmic reticulum	987	^82^, ^83^
CBU1376	CoxK2	None	1365	^43^
CBU1379	None	Predicted Ser/Thr kinases	849	^70^
CBU1387	EmcA	Inhibiting RIG‐I signaling and the production of type I IFNs	651	^81^
CBU1406	None	None	833	^59^
CBU1409	None	None	837	^68^
CBU1425	MceC	Interacting with components of the mitochondrial quality control machinery	453	^79^
CBU1434	None	None	462	^59^
CBU1457	Cig43	None	2025	^59^, ^70^
CBU1460	None	None	786	^64^
CBU1461	CoxCC8	None	2270	^43^
CBUD1532	CaeB	Inhibiting MOMP after Bax/Bak activation, thereby suppressing intrinsic apoptosis	711	^84^
CBUD1524	CaeA	Preventing the cleavage of caspase 7 without affecting the activation of caspase 9.	3510	^85^
CBU1525	None	None	3028	^64^
CBU1543	MceF	Recruiting GPX4 to the mitochondria	567	^86^
CBU1556	CvpC	None	1704	^87^
CBU1566	None	None	735	^59^
CBU1569	CoxCC12	None	1644	^43^
CBU1576	None	None	1965	^59^
CBU1594	MceD	None	450	^79^
CBUD1599	None	None	2520	^59^
CBU1607	None	None	399	^59^
CBUD1620	None	None	696	^59^
CBU1634a	None	None	150	^68^
CBU1639	None	None	1197	^64^
CBU1665	None	None	1740	^64^
CBU1676	None	Modulation of the MAPK pathway	1086	^68^
CBU1677	MceE	None	435	^79^
CBU1724	None	Encoding a predicted F‐box protein	2244	^70^
CBU1751	Cig57	Recruiting clathrin to CCV in a manner dependent on FCHO2	1263	^88^
CBU1752	None	None	1239	^60^
CBU1754	None	None	597	^59^
CBUD1776	None	None	2115	^54^
CBU1780	None	None	1590	^43^
CBU1789	None	None	867	^59^
CBU1794	None	None	819	^68^
CBU1799	None	None	462	^70^
CBU1818	CvpD	None	1446	^87^
CBU1823	IcaA	Inhibiting Caspase‐11‐mediated NLRP3 inflammasome activation	1050	^89^
CBU1825	None	None	348	^90^
CBU1863	CvpE	Interacting with lysosome‐derived PI(3)P on CCV, interfering with PIKfyve activity, and inhibiting the formation of lysosome tubulation	1812	^91^
CBUD1963	None	None	966	^54^
CBU2007	None	None	1188	^90^
CBU2013	EmcB	Removing the K63‐linked ubiquitin chains on RIG‐I	1098	^81^
CBU2016	None	None	597	^64^
CBU2028	None	None	747	^60^
CBU2052	CirD	None	903	^59^
CBU2056	None	None	663	^43^
CBU2059	CirE	None	1592	^59^
CBU2072	EirA	None	498	^92^
CBU2078	None	None	1047	^64^
CBU2076	None	None	318	^59^
CBU2082	None	None	768	^43^

CBU, *Coxiella burnetii* RSA 493; CBUA, *C. burnetii* RSA 493 pQpH1; CBUD, *C. burnetii* Dugway 5J108‐111.

The development of axenic culture techniques and *Himar1* transposon mutagenesis has greatly contributed to our understanding of the roles that *C. burnetii* T4Es play in manipulating host processes and facilitating the formation of the CCV. Recently, the toolbox was expanded with the introduction of a CRISPR‐Cas9‐mediated cytosine‐based editing method, enabling precise gene targeting. This new approach will be invaluable for dissecting potential synergy among T4E genes by creating and analyzing mutants lacking multiple genes^53^. In the following sections, we will explore the mechanism of action of effectors that modulate key host cell processes, including clathrin‐mediated vesicle trafficking, autophagy, apoptosis, pyroptosis, and immune signaling.

## MODULATION OF CLATHRIN‐MEDIATED VESICLE TRAFFICKING

Endocytosis is a key process in eukaryotic cells that facilitates the transport of various cargoes across the plasma membrane into the cytoplasm. These cargoes primarily consist of transmembrane proteins and their extracellular ligands, which play key roles in a wide range of physiological processes. Clathrin‐mediated endocytosis, as a form of receptor‐mediated endocytosis, serves as a primary pathway for the internalization of the cargoes^93^.

Endocytosis begins with the aggregation of initial endocytic proteins on the plasma membrane, forming the core for the assembly of the vesicle coat^93^. Early studies have demonstrated that, in mammalian cells, the BAR domain proteins F‐BAR domain only protein 1 (FCHO1) and FCHO2, as well as the AP2 complex, may act as the primary initiators^94^. These adapters, FCHO1/2 and AP2, bind to the plasma membrane and recruit scaffold proteins, subsequently aggregating membrane‐bound adapters to form endocytic sites^95^. The basic principle of cargo recruitment involves the protein components of the clathrin coat engaging specific binding sites on the cytoplasmic portions of transmembrane cargo molecules, recruiting them to the plasma membrane region where vesicles will form. This process results in the enrichment of specific cargo in the forming vesicles, leading to selective endocytosis. Many clathrin‐associated adapters and scaffold proteins have been shown to interact directly with specific cargo and serve as their cargo adapters. This includes several early coat components, such as the adapter protein AP2, FCHO1, EPS15, and CALM family^96^. Therefore, cargo molecules may recruit these adapter proteins to the plasma membrane, increasing the likelihood of initiating endocytic events^97^. In addition to binding to cargo, most cargo adapters also interact directly with lipids and other membrane proteins, forming a complex interaction network that mediates the assembly and further extension of the clathrin coat in a highly intricate and collaborative manner^98^. The AP2 complex plays a crucial role in both initiation and maturation process of clathrin‐coated pits through an allosteric regulation mechanism. Specifically, the AP2 complex is activated when its α and β2 subunits bind to PI(4,5)P2 and cargo containing the Yxxφ motif, triggering clathrin polymerization at the plasma membrane and initiating the formation of clathrin‐coated pits. Subsequently, the activated AP2 complexes on the plasma membrane are further stabilized through their µ2 subunit binding to PI(4,5)P2 or by phosphorylation of the µ2 subunit at T156. This stabilization is further regulated by clathrin assembly through an AAK1‐dependent feedback loop, which promotes clathrin polymerization. Continuous interactions between PI(4,5)P2 and the binding sites on α, β2, and µ2 are essential for the stabilization and efficient maturation of clathrin‐coated pits^99^. The assembled clathrin coat induces membrane curvature, transforming the flat plasma membrane into a “clathrin‐coated pit”. The scission process then constricts and separates the neck of the membrane invagination, detaching the clathrin‐coated vesicle from the plasma membrane. Actin polymerization works in concert with the coat and scission proteins to facilitate membrane shape changes. Finally, during uncoating, the endocytic machinery disassembles, releasing cargo‐filled nascent vesicles for fusion with early endosomes (EEs), thus initiating intracellular transport cascades. The released endocytic proteins can then be reused for another endocytic event^93^.

After the disassembly of clathrin coats, newly formed endocytic vesicles fuse with one another and with sorting endosomes. EEs consist of two distinct organelles: sorting endosome (SE) and recycling endosomes (RE)^100^. The SE serves as the primary branching point in the receptor‐mediated endocytic pathway, directing molecules to their appropriate destinations. Beyond the SE, there are three primary destinations: the plasma membrane, late endosomes (LEs), and the RE. There are two primary pathways from the SE to the cell surface: some recycling molecules are directly returned to the plasma membrane, while others are directed to RE, which is a long‐lived organelle. The RE primarily consists of a network of tubular organelles, approximately 60 nm in diameter, and is closely associated with microtubules^101^, ^102^, ^103^. The RE plays a critical role in sorting molecules to various destinations, with the majority of molecules within the RE ultimately being recycled back to the plasma membrane.

*C. burnetii* actively recruits certain clathrin‐coated vesicles to the CCVs, enabling it to separate the outer membrane from the clathrin‐mediated vesicular transport network. This process facilitates the transfer of nutrients, including cargo proteins and lipids, from the vesicles to the CCVs, promoting their expansion to support intracellular bacterial replication. This conclusion is supported by observations of clathrin accumulation on the CCVs^77^, ^88^. Furthermore, silencing the genes encoding clathrin or the clathrin adapter protein AP2 significantly impairs CCV expansion and *C*. *burnetii* replication, highlighting its crucial role in the intracellular life cycle of the bacterium^77^, ^104^. Subsequent studies have shown that silencing of the *FCHO2* gene or *CLTC* results in the formation of smaller CCVs with a multivesicular phenotype, further supporting the idea that *FCHO2* and *CLTC* are essential for forming normal CCV^88^.

CvpA interferes with the acquisition of endolysosomal lipids and proteins associated with clathrin‐coated vesicles. It was identified through bioinformatics analysis of the *C*. *burnetii* Nine Mile RSA493 genome. This effector contains a leucine‐rich repeat (LRR) and several endocytic sorting motifs ([DERQ]XXXL[LI] and YXXΦ), which resemble motifs recognized by the clathrin adapter protein complexes AP1, AP2, and AP3^105^. CvpA binds to membrane‐bound AP2 via its endocytic sorting motifs, and deletion of *cvpA* leads to significant defects in bacterial intracellular growth. Similarly, silencing of *AP2* or *CLTC* with siRNA leads to reduced intracellular replication of *C*. *burnetii* and smaller CCVs, indicating that CvpA promotes CCV development and intracellular replication, probably by hijacking the AP2‐clathrin vesicle transport pathway. Live cell imaging using fluorescence microscopy revealed that ectopically expressed CvpA appears to be dynamically transported via peripheral tubular vesicles and concentrates in a cluster of polymorphic tubules and vesicles in the pericentrosomal region of the cell. During *C*. *burnetii* infection, CvpA localizes to CCVs. Additionally, ectopically expressed CvpA colocalizes with the small GTPase Rab5 at the cell periphery and significantly colocalizes with Rab11 around the pericentrosome. This suggests that CvpA is trafficked to peripheral SE and pericentrosomal RE, where endocytic cargo is sorted and either delivered to other vesicular compartments or recycled back to the cell surface. These observations indicate that CvpA traffics within the endocytic recycling compartment and localizes to the CCV membrane (Figure 2).

The transferrin receptor (TfR) is transported through pericentrosomal RE, eventually being returned to the cell surface in a process that requires Rab11^106^, ^107^, ^108^. Disruption of Rab11 or AP2 impairs TfR endocytosis and its subsequent trafficking^106^, ^108^, ^109^, ^110^. To further investigate the role of CvpA in endosomal trafficking, researchers treated cells expressing CvpA with Brefeldin A (BFA), which disrupts vesicle coats, resulting in the formation of tubular structures in endosomes and fragmentation of Golgi stacks^111^. Ectopically expressed CvpA colocalized with TfR in tubular endosomes at the peripheral region of the cell, further suggesting that CvpA is involved in endosomal recycling^77^. Additionally, ectopically expressed CvpA impaired the uptake of fluorescently labeled transferrin (Tf) by HeLa cells, suggesting that this effector disrupts Tf trafficking^77^.

Cig57 is another *C*. *burnetti* T4E that co‐opts the clathrin‐mediated vesicle transport pathway^88^ (Figure 2). Cig57 interacts with FCHO2 (Figure 2), a component of clathrin‐coated vesicles, to recruit these vesicles to the CCV^77^. This process may provide membrane materials for the expansion of CCV and facilitate the transfer of nutrients, in the form of cargo proteins and lipids, to the CCV. FCHO2 is believed to play a key role in the initiation of clathrin‐mediated endocytosis, arriving early at the clathrin‐mediated endocytic sites and assisting in shaping the cell membrane to form spherical clathrin‐coated vesicles^112^, ^113^ (Figure 2). The N‐terminus of FCHO2 contains an EFC domain responsible for membrane binding, dimerization, and inducing membrane curvature, along with a connector region and a C‐terminal μ‐homology domain^114^. Yeast two‐hybrid assays have shown that Cig57 interacts with the N‐terminal region of FCHO2 (amino acids 1–433) via its tyrosine‐based endocytic sorting motif. This suggests that Cig57 may influence the membrane‐binding capacity of FCHO2 by preventing its dimerization, altering membrane curvature, or affecting posttranslational modifications. The ∆*cig57* mutant is unable to recruit clathrin to the CCV, and similarly, clathrin recruitment is reduced in cells lacking FCHO2. These findings indicate that recruitment of clathrin by Cig57 occurs in a manner that requires FCHO2^88^ (Figure 2).

## MODULATION OF AUTOPHAGY

Autophagy is a highly regulated catabolic process that sequesters intracellular materials within autophagosomes, which are then transported to lysosomes for degradation. This process not only facilitates nutrient recycling, supporting cell survival under starvation conditions, but also enables cells to capture and transport damaged organelles, misfolded proteins, and certain intracellular pathogens to lysosomes for degradation. As such, autophagy plays a crucial role in maintaining cellular homeostasis.

Delivering invading pathogens to the lysosomal compartment via autophagy is a crucial mechanism of cellular defense^115^. The cytoplasmic surface of phagosomes containing certain pathogens exhibits characteristics similar to those of damaged organelles, allowing them to be recognized and modified by the host's ubiquitination machinery. These ubiquitin signals are then recognized by autophagy receptors, triggering the initiation of a selective form of autophagy known as “xenophagy”. This process is characterized by the formation of a membrane enriched with Atg8‐like proteins, such as LC3^116^, ^117^. Inhibition of xenophagy by intracellular bacterial pathogens is well documented^118^. For example, the SidE family effectors of *L*. *pneumophila* prevent the recognition of autophagy adapter proteins by forming unique, noncanonical ubiquitin chains on bacterial phagosome. When all four SidE family members (SidE, SdeA, SdeB, and SdeC) are deleted, the recruitment of Sequestosome‐1 (SQSTM‐1) to the bacterial vacuole significantly increases^119^, ^120^.

**Figure 3:**
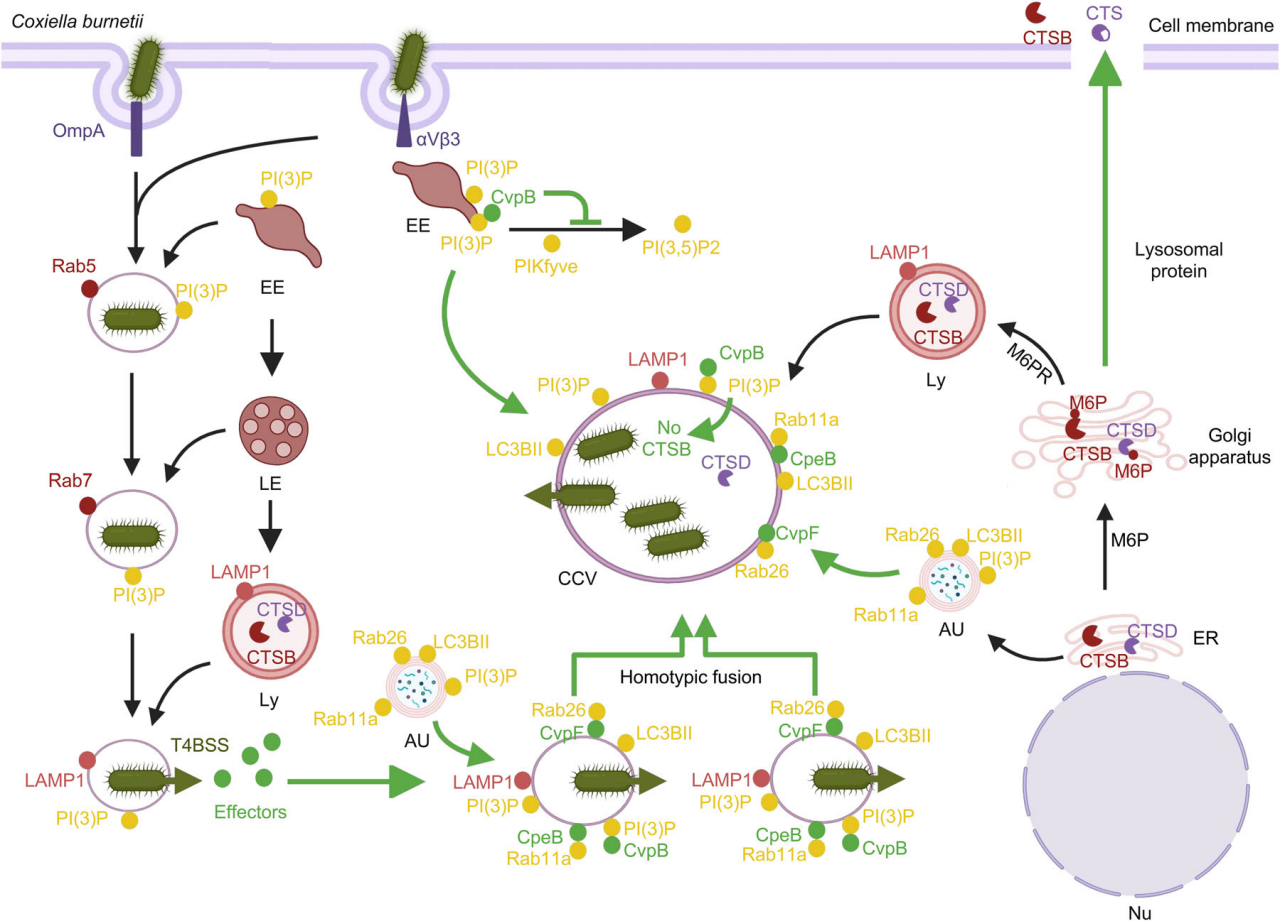
Modulation of host cell autophagy by *C. burnetii* Dot/Icm effectors. The effector CvpB binds to PI(3)P on CCVs and EEs, enriching PI(3)P on these subcellular structures and facilitating the fusion of EEs with the CCVs, as well as homotypic fusion between CCVs. CvpB also modulates the lysosomal environment by affecting the formation of CCVs, leading to the clearance of cathepsin B (CTSB) from the host cell. Furthermore, *C. burnetii* infection can induce the secretion of lysosomal proteins, including pro‐cathepsin B and D (CTSB and CTSD), from the Golgi apparatus to the extracellular space. The effector CvpF localizes to vacuoles with autolysosomal characteristics and CCVs, where it anchors and/or activates Rab26 on EEs and CCVs. This action disrupts Rab26‐dependent autophagy and stimulates the accumulation of LC3BII on the CCVs, ultimately promoting the maturation into an autolysosomal state. Additionally, CpeB interacts with the autophagy‐related protein Rab11a, promoting the accumulation of LC3BII. Created in BioRender. Song, L. (2025) https://BioRender.com/kb3ce71.

Within a few hours of entry, fusion between the CCV and autophagosomes occurs, a process that requires *de novo* protein synthesis by *C*. *burnetii* ^121^, ^122^. The multifunctional T4E Cig2/CvpB is essential for the homotypic fusion of CCVs and the recruitment of LC3 to the bacterial phagosome. A *C. burnetii* mutant lacking *cig2* exhibited a multivesicular phenotype^65^, similar to the phenotype observed in cells defective in autophagy that were infected by wild‐type bacteria^43^. Cells infected with the ∆*cig2* mutant also showed a reduction in LC3 accumulation on the bacterial vacuole, indicating that this effector is crucial for both the homotypic fusion of CCVs and LC3 recruitment (Figure 3). The formation of autophagolysosome is essential for homotypic fusion of CCVs, which generates large CCVs required for optimal *C*. *burnetii* replication^66^, ^123^. The cellular targets of Cig2 and the biochemical basis of its modulation of these targets remain unknown. Since the fusion of autophagosomes with lysosome‐derived vesicles is a classical pathway for delivering autophagy cargo to the lysosome, it is speculated that Cig2 may interfere with the steady‐state process that regulates fusion between autophagosomes.

Ectopically expressed CvpB/Cig2 localizes to EEs through interaction with PI(3)P, and most CvpB/Cig2 is recruited to CCVs in infected cells^66^ (Figure 3). The C‐terminal domain of CvpB interferes with the recruitment of the PI5‐kinase PIKfyve to PI(3)P‐positive membranes, disrupting its function and leading to the accumulation of PI(3)P in specific subcellular compartments (Figure 3). In EEs, the accumulation of PI(3)P induces vacuolation and clustering of EEs, which facilitates in the delivery of these endosomal structures to CCVs and supports optimal CCV expansion (Figure 3). At CCVs, CvpB promotes the association of PI(3)P on CCVs by inhibiting the kinase activity of PIKfyve, thereby stabilizing the autophagy machinery responsible for homotypic fusion between CCVs^66^ (Figure 3).

A recent study revealed that CvpB is also involved in the removal of the lysosomal cysteine protease cathepsin B from the infected cells. Overexpression of cathepsin B is detrimental to intracellular replication of *C. burnetii* and the development of CCVs, while cells infected with *C. burnetii* exhibit a loss of its activity^124^. Cathepsin B is removed from infected cells through two distinct mechanisms. The first involves CvpB, which influences CCV formation, alters the lysosomal environment, and ultimately facilitates the removal of cathepsin B from infected cells (Figure 3). Intriguingly, *C. burnetii* infection stimulates the secretion of lysosomal proteins, including pro‐cathepsin B, from the Golgi apparatus into the extracellular space. This process can be completely inhibited by BFA, a classical drug that blocks the assembly of COP‐I coats by targeting the small GTPase Arf1^124^, ^125^, ^126^. Another T4E, CvpE, interacts with lysosome‐derived PI(3)P on CCV and lysosomes, interfering with PIKfyve activity and impairing the generation of PI(3,5)P2. This disruption of PI(3,5)P2 generation leads to defects in lysosomal fission and enlargement by blocking the PI(3,5)P2‐TRPML1‐Ca^2+^ signaling pathway, which, in turn, promotes the enlargement of the CCV^91^.

CvpF interacts with and recruits Rab26 to CCVs, promoting the formation of the lipidated form of LC3 (LC3BII) and its accumulation on CCVs^76^ (Figure 3). Ectopically expressed CvpF colocalizes with the endosomal sorting complex required for transport complex, lysosomes, and autophagosomes. In cells infected with *C. burnetii*, CvpF colocalizes with the lysosomal marker LAMP1 on CCVs. These findings suggest that CvpF plays a crucial role in the formation of CCVs and the rerouting of autophagosomes. Interestingly, when autophagic flux was blocked using bafilomycin A1, the expression of CvpF still resulted in an increase in LC3BII levels. Moreover, starvation of cells expressing CvpF did not affect SQSTM degradation, indicating that the accumulation of LC3BII on CCVs induced by this effector was not due to stimulation of autophagic flux.

CvpF preferentially binds to the inactive form of Rab26, but only wild‐type Rab26 is targeted to membranes^76^. These observations suggest that CvpF functions as a guanine nucleotide exchange factor (GEF) or a GDI displacement factor (GDF), activating and anchoring Rab26 to CCVs. The active form of Rab26 has been shown to interact with ATG16L1, a protein essential for the anchoring of LC3BII to membranes^127^. Cells expressing the inactive form of Rab26 exhibited a significant reduction in the recruitment of LC3BII to CCVs, along with a decrease in CCV size. Additionally, knockdown of Rab26 resulted in smaller CCVs and a significant reduction in bacterial replication^76^. Thus, Rab26‐dependent autophagy is crucial for the recruitment of LC3BII to the CCV, which is important for its development.

The *Coxiella* effector CpeB, encoded by the plasmid QpH1, also localizes to the CCV and promotes the accumulation of LC3BII on the bacterial phagosome^128^ (Figure 3). *C*. *burnetii* strains lacking QpH1 exhibit reduced LC3BII accumulation, form smaller CCVs, and exhibit a lower bacterial load in THP‐1 cells^63^. Interestingly, expression of CpeB in QpH1‐defective strains restores LC3BII accumulation but does not rescue the small CCV phenotype, suggesting that CpeB coordinates with other effectors to promote the formation of larger CCVs^128^. CpeB interacts with Rab11a, a protein known to play a role in the biogenesis of autophagosomes through interacting with WIPI2 and PI(3)P^129^. Knockdown of Rab11a suppresses CpeB‐induced LC3BII production^128^, indicating that Rab11a plays a role in CpeB‐mediated LC3B accumulation on the CCV.

Among the three *C. burentii* effectors, i.e., Cig2, CvpF, and CpeB, that are currently known to modulate autophagy, the biochemical mechanism by which Cig2 inhibits PIKfyve kinase activity, thereby enriching PI(3)P on CCVs, remains unclear. CvpF preferentially interacts with the inactive form of Rab26, promoting its association with endosomes, suggesting that it functions as a GDI or GEF for the small GTPase. It has been shown that CCVs are decorated with Rab1, Rab7, and Rab24^130^, ^131^, ^132^, and it is likely that this association is mediated by one or more T4Es. Although *C. burnetii* infection leads to an increase of LC3BII in host cells, the overall autophagic flux rate does not increase^43^, ^121^, ^122^. Since CvpF appears to stimulate autophagic flux, this raises the possibility that other effectors may balance its effects by blocking autophagic flux. In addition, recent studies have found that CstK, a key protein kinase of *C. burnetii*, which can autophosphorylate itself on Thr and Tyr residues, also phosphorylates typical eukaryotic protein kinase substrates in biochemical reactions^72^. CstK interacts with the host protein TBC1D5, and during *C. burnetii* infection, CstK is anchored to the surface of the CCV, where it recruits TBC1D5 to these vacuoles. However, phosphorylation of TBC1D5 by CstK has not been detected. TBC1D5 interacts with LC3 and VPS29, acting as a molecular switch between the endocytic and autophagy pathways. Therefore, CstK may promote bacterial replication by influencing both the endocytic and autophagy pathways^72^.

## MANIPULATION OF HOST CELL DEATH PATHWAYS BY *C*. *burnetii*

Apoptosis is a form of programmed cell death triggered by the activation of a cascade of caspases^133^, and it plays a critical role in innate immunity^134^. During infection, apoptosis enables the host to eliminate infected cells, thereby removing the niche that supports pathogen replication^135^. In some cases, the induction of cell death also triggers inflammation, further enhancing the protective immune response^136^.

**Figure 4:**
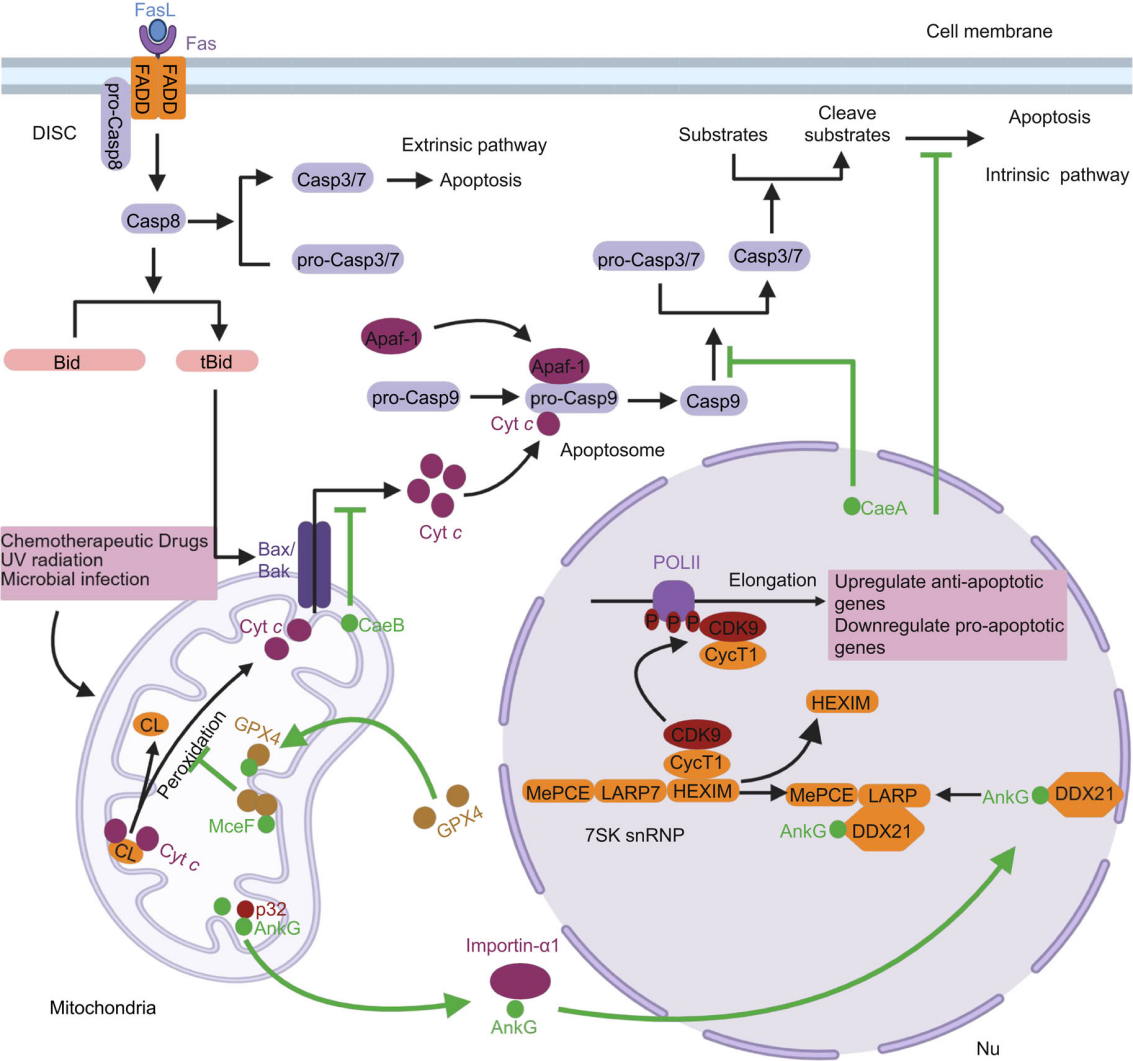
Manipulation of host cell apoptosis by *C. burnetii* Dot/Icm effectors. Under stress conditions, AnkG is translocated from the mitochondrion to the nucleus with the assistance of p32, where it interacts with DDX21 and 7SK snRNP to regulate the expression of genes involved in apoptosis, cell trafficking, and transcription. This interaction helps inhibit infection‐induced apoptosis. CaeA localizes to the nucleus, where it inhibits both intrinsic and extrinsic apoptosis by preventing the activation of the effector Caspase‐7 (Casp7), without affecting the cleavage of the initiator Casp9. CaeB localizes to the mitochondria or the ER, where it primarily suppresses mitochondrial outer membrane permeabilization (MOMP) following Bax/Bak activation, thereby inhibiting intrinsic apoptosis. MceF recruits GPX4 to the mitochondrial inner membrane, where it depletes excessive mitochondrial ROS (mROS) in the mitochondria, inhibiting the peroxidation of cardiolipin (CL) and the release of Cyt *c* associated with CL, thereby suppressing cell apoptosis. CycT1, cyclin T1; DDX21, DExD box RNA helicase 21; DISC, death‐inducing signaling complex; snRNP, small nuclear ribonucleoprotein; t‐BID, truncated BID. Created in BioRender. Song, L. (2025) https://BioRender.com/yo6obd3.

Apoptosis can be initiated through either the intrinsic or extrinsic pathway. The intrinsic pathway is triggered by the release of apoptosis‐inducing proteins, such as cytochrome *c*, SMAC/Diablo, and HtrA2/Omi, from the mitochondrial intermembrane space into the cytoplasm in response to various stresses, including chemotherapeutic drugs, UV radiation, and microbial infection^137^ (Figure 4). Once released, cytochrome *c* binds to apoptotic protease‐activating factor‐1 (Apaf‐1), forming an apoptosome complex that activates Caspase‐9, the initiator caspase^138^ (Figure 4). Activated Caspase‐9 subsequently cleaves and activates effector caspases (Caspase‐3 and ‐7) by removing their inhibitory prodomain (Figure 4). Executioner caspases require release from XIAP (X‐linked inhibitor of apoptosis protein) to fully exert their function (Figure 4). The mitochondrial death proteins HtrA2/Omi and SMAC/Diablo facilitate this release by binding to XIAP through their N‐terminal IAP binding motif (IBM)^137^. Once released, these mature caspases cleave various substrates, such as PARP‐1 and ICAD, leading to typical apoptotic changes, including DNA fragmentation^139^, ^140^, ^141^.

The extrinsic apoptosis pathway is initiated by the binding of ligands, such as tumor necrosis factor (TNF) and Fas ligand (FasL or CD95L), to death receptors, leading to the formation of the death‐inducing signaling complex (DISC). This complex contains pro‐caspase‐8 and adapter proteins FADD/TRADD^142^, ^143^, ^144^, ^145^ (Figure 4). Activation of Caspase‐8 can trigger apoptosis either directly by activating Caspase‐3 and Caspase‐7^146^, or indirectly by cleaving BID into truncated BID (t‐BID), which then promotes mitochondrial apoptosis^147^, ^148^ (Figure 4).

Induction of apoptosis can limit microbial replication and restrict pathogen spread. For instance, the induction of intrinsic apoptosis in dendritic cells has been shown to reduce replication of *L*. *pneumophila* ^149^. In contrast, defects or delays in the induction of apoptosis can exacerbate disease progression and lead to significant tissue damage. For example, in a pneumococcal pneumonia disease model, macrophages in transgenic mice overexpressing Mcl‐1, an antiapoptotic member of the Bcl‐2 family, fail to effectively clear bacteria from the lungs^150^. However, apoptosis induction is not always protective for the host, as some viruses and bacteria can exploit the host's apoptosis machinery to eliminate antiviral cells. For example, the rapid depletion of lymphocytes in HIV patients^151^, ^152^ and the apoptotic destruction of circulating lymphocytes in septic shock patients^153^, ^154^ are both associated with poor clinical outcomes.

*C*. *burnetii* inhibits apoptosis through the activity of several T4Es, and this inhibition promotes its survival and replication within host cells^64^, ^84^. Among these, AnkG normally localizes to the mitochondria under normal conditions but must translocate to the nucleus to exert its antiapoptotic effect^155^ (Figure 4). This effector interacts with the host protein p32 through its N‐terminal arginine‐rich region (residues 19‐24) and is transported to the nucleus with the assistance of stress‐induced p32^155^, ^156^ (Figure 4). The nuclear import of AnkG requires the binding of its N‐terminal nonclassical nuclear localization signal (aNLS) to importin‐α1^157^ (Figure 4). Once in the nucleus, AnkG interacts with the host cell's DExD box RNA helicase 21 (DDX21) and the 7SK small nuclear ribonucleoprotein (7SK snRNP) complex, facilitating the release of the transcription elongation factor b (P‐TEFb) from the 7SK snRNP complex. P‐TEFb, composed of CDK9 and Cyclin T1 (CycT1), then phosphorylates several transcriptional regulators, including NELF, DSIF, and the C‐terminal domain of RNA polymerase II (Figure 4). These promoter‐proximal phosphorylation events enable RNA polymerase II to resume transcription, thereby regulating the expression of genes involved in apoptosis, cell trafficking, and transcription, which plays a crucial role in inhibiting infection‐induced apoptosis^157^.

CaeA localizes to the nucleus when ectopically expressed in mammalian cells^54^ (Figure 4). It inhibits both intrinsic and extrinsic apoptosis by preventing the activation of the executioner Caspase‐7, without affecting the cleavage of initiator Caspase‐9^85^ (Figure 4). Additionally, CaeA increases the protein levels of survivin, though this effect is independent of its antiapoptotic activity^85^. A sequence comparison of CaeA alleles from 25 different *C*. *burnetii* isolates revealed a critical EK (glutamate/lysine) short tandem repeat motif. CaeA variants containing 3, 4, or 6 copies of the EK repeat are essential for its antiapoptotic activity, although the precise mechanism underlying the activity of these repeats remains elusive^85^.

CaeB localizes to both mitochondria^54^ and the endoplasmic reticulum (ER)^71^, where it exerts an inhibitory effect on apoptosis (Figure 4). CaeB inhibits mitochondrial outer membrane permeabilization (MOMP) without interfering with the mitochondrial targeting of Bax or altering the steady‐state levels of antiapoptotic Bcl‐2 family proteins, including Bcl‐2, Bcl‐XL, and Mcl‐1. It primarily inhibits MOMP after Bax/Bak activation, thereby suppressing intrinsic apoptosis^158^ (Figure 4). Notably, CaeB does not interact with p32, suggesting that, unlike AnkG, it does not interfere with apoptosis by binding to proapoptotic mitochondrial proteins. The precise mechanism by which CaeB inhibits mitochondrial outer membrane permeabilization remains to be fully elucidated.

As the infection progresses to its late stage, nutrients in infected cells become depleted, and bacteria must be released to initiate a new round of infection. This process may be associated with apoptosis induced by the bacterium^159^. Activation of the interferon gene‐stimulating factor (STING) signaling pathway plays a role in the induction of apoptosis^160^. Recognition of cytosolic bacterial DNA activates the cGAS‐cGAMP‐STING‐IRF3 signaling axis^161^. The binding of cytoplasmic IRF3 to BAX promotes its translocation to the mitochondrial membrane, leading to mitochondrial membrane depolarization and the release of mitochondrial DNA and cytochrome *c* into the cytoplasm. This, in turn, further activates cGAS, establishing a positive feedback loop that amplifies the STING signaling pathway and exacerbates mitochondrial damage^160^. Additionally, released cytochrome *c* activates Caspase‐9, triggering apoptosis, which coincides with bacterial release^160^.

Pyroptosis is an inflammatory form of programmed cell death that can be categorized into two types: the canonical form, which is Caspase‐1‐dependent, and the noncanonical form, which occurs independently of Caspase‐1^158^, ^162^. The classical Caspase‐1‐dependent pyroptosis pathway is typically triggered by the recognition of pathogen‐associated molecular patterns (PAMPs) or damage‐associated molecular patterns (DAMPs) by an inflammasome or its sensors. Activation of the inflammasome leads to the formation of a multiprotein complex that facilitates the self‐processing and activation of Caspase‐1^162^, ^163^, ^164^, ^165^, ^166^.

**Figure 5:**
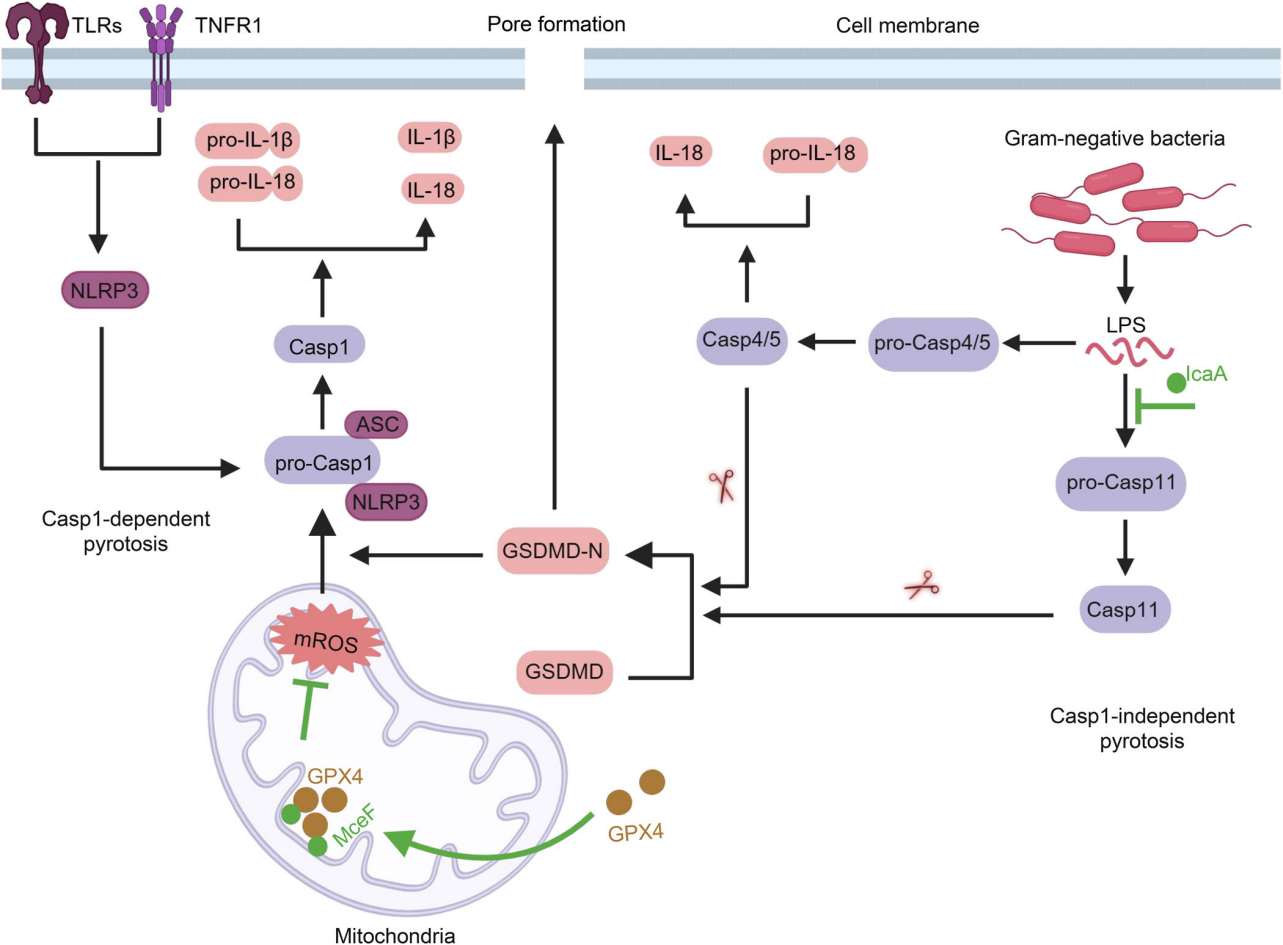
Manipulation of host cell pyroptosis by *C. burnetii* Dot/Icm effectors. IcaA inhibits the noncanonical activation of the NLRP3 inflammasome by suppressing activation of Casp11. MceF can inhibit the activation of the NLRP3 inflammasome and the cleavage of Gasdermin D (GSDMD) induced by mROS by recruiting GPX4 to the inner membrane of mitochondria, thereby depleting mROS levels, ultimately inhibiting pyroptosis. Created in BioRender. Song, L. (2025) https://BioRender.com/ha68vsz.

The noncanonical Caspase‐1‐independent pyroptosis pathway is activated by LPS released by invading bacteria, which is sensed by Caspase‐4/5 (in humans) or Caspase‐11 (in mice) (Figure 5). Engagement of LPS causes autocleavage and activation of these caspases, which then proteolytically process Gasdermin D (GSDMD) to execute pyroptosis^158^, ^162^. Activated human Caspase‐4 also directly facilitates the maturation of IL‐18^167^ (Figure 5).

The *C*. *burnetii* NMII strain induces Caspase‐1‐dependent pyroptosis in murine peritoneal B1a cells through a process that requires the Dot/Icm system, as well as the activation of TLR‐2 and NLRP3. In contrast, the virulent NMI strain lacks this ability^115^. These two strains differ in the length of their LPS^168^, which may account for the difference in this phenotype.

*L. pneumophila* induces flagellin‐independent pyroptosis through a process that requires Caspase‐11^169^. In contrast, *C. burnetii* does not induce pyroptosis in infected bone‐marrow‐derived macrophages (BMDMs). However, transfecting BMDMs with *C. burnetii* extract enriched in LPS can induce caspase‐11‐dependent pore formation^89^. Given that studies have shown tetra‐acylated lipid A cannot activate caspase‐11^170^, this suggests that lipid A of *C. burnetii* undergoes changes during different stages of its infection. It is also possible that *C. burnetii* possesses other molecules capable of activating caspase‐11. By expressing *C. burnetii* genes in an *L*. *pneumophila* strain lacking flagellin and testing the resulting strains for the ability to induce Caspase‐1 activation, Cunha et al. found that the T4E IcaA effectively inhibits *L. pneumophila*‐induced inflammasome activation^89^. Co‐infection experiments revealed that the *icaA*‐deficient mutant of *C*. *burnetii* lost the ability to inhibit Caspase‐11‐mediated inflammasome activation induced by *L*. *pneumophila*, suggesting that IcaA plays a role in inhibiting Caspase‐11‐mediated noncanonical inflammasome activation^89^. Pyroptotic cell death not only eliminates the niche required for intracellular pathogen to replicate but also releases inflammatory cytokines that recruit and activate immune cells, leading to further pathogen clearance^162^. IcaA clearly plays a role in maintaining the niche for *C*. *burnetiid* growth. Future studies aiming at elucidating its mechanism of action will provide valuable insights into both its biochemical activity and potentially novel regulation mechanisms of innate immunity.

Finally, *C. burnetii* utilizes the effector MceF to co‐opt host Glutathione Peroxidase 4 (GPX4) to inhibit oxidative stress‐induced cell death^86^. MceF is targeted to the mitochondrial inner membrane, where it specifically recruits GPX4 to reduce the production of mitochondrial ROS (mROS) induced by agents such as rotenone, thereby preventing subsequent cell death (Figures 4 and 5). The removal of mROS by MceF may also inhibit the activation of the NLRP3 inflammasome (Figure 5). To date, MceB, MceC, MceD, and MceE are the four known *C. burnetii* effectors that target the mitochondrion^79^. Among them, MceC is associated with the mitochondrial inner membrane, where it interacts with components of the mitochondrial quality control machinery, suggesting that it may play a role in regulating mitochondrial function. The function of MceB remains unknown. Although it is homologous to bacterial porins, it lacks the classical β‐targeting signals typical of mitochondrial β‐barrel proteins, and its specific function has yet to be determined^79^. A more recent study indicated that MceB is localized to the outer membrane of *C. burnetii* ^80^.

## REGULATION OF NFκB SIGNALING

The transcriptional factor (NFκB) regulates the expression of a wide range of genes involved in various cellular functions, including immunity, cell proliferation, and apoptosis^171^. Pathogens have evolved various strategies to modulate NFκB activity by targeting different points in the signaling cascade^172^, ^173^. Infection with *C*. *burnetii* strongly induces NFκB activation, but this activation appears to be suppressed as infection proceeds, likely due to the activity of one or more T4Es^174^. Inhibition of NFκB signaling after the initial phase of infection is consistent with the fact that the transporter does not become active until at least 4–8 h after bacterial uptake^50^, ^175^.

**Figure 6:**
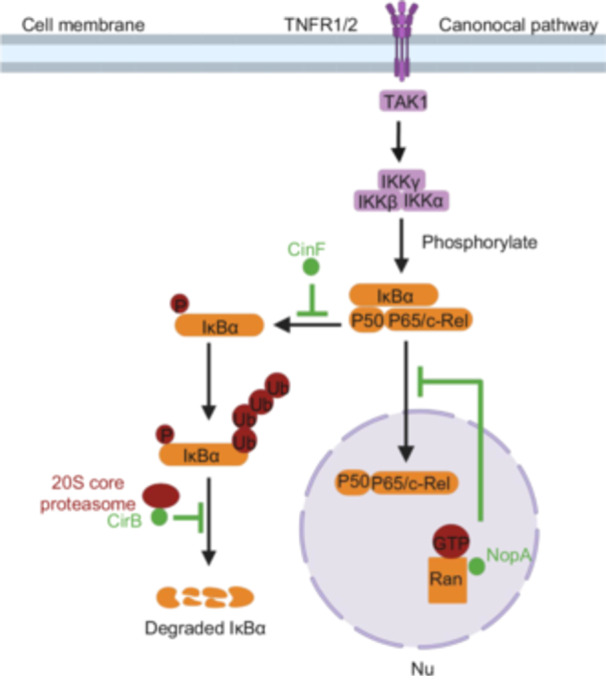
Regulation of the NF‐κB signaling pathway by *C. burnetii* Dot/Icm effectors. NopA prevents the nuclear translocation of the NF‐κB by directly interacting with the Ran GTPase, thereby interfering with its nuclear import. CinF functions as a protein phosphatase, dephosphorylating IκBα and making it resistant to proteasome‐mediated degradation, which blocks the exposure of the nuclear localization signal of NF‐κB, thereby inhibiting its activity. CirB interacts with the 20S core proteasome, inhibiting its hydrolytic activity and preventing the degradation of IκBα. Created in BioRender. Song, L. (2025) https://BioRender.com/gf6vi3s.

Two T4Es have been identified as inhibitors of NFκB signaling. One of these effectors, NopA, is associated with nucleoli of infected cells, where it interacts with the Ran GTPase via an RCC‐like domain localized in its C‐terminal region. Notably, NopA preferentially binds to the inactive Ran‐GDP^70^ (Figure 6). This interaction promotes the accumulation of Ran‐GTP in the nucleolus, which subsequently interferes with the nuclear import of NFκB (Figure 6). However, it remains unclear whether binding alone is sufficient for NopA to exert its effects^70^. It is possible that NopA induces posttranslational modifications on Ran to inhibit its activity.

CinF is another *Coxiella* effector that inhibits NFκB activation. It functions as a protein phosphatase by dephosphorylating IκBα, rendering it resistant to ubiquitination and subsequent degradation by the proteasome^75^ (Figure 6). Importantly, loss of *cinF* caused a significant defect in intracellular bacterial growth^60^, ^75^. Intriguingly, CinF shares significant similarity (approximately 39% identity and 65% similarity) with TnFBPAP from *Thermoproteus neutrophilus*, a fructose‐1,6‐bisphosphate (FBP) aldolase/phosphatases involved in glycogenesis^75^. The connection between metabolism and immune response has been well established^176^, and this discovery suggests that metabolic enzymes can directly influence the immune signaling pathway. In support of this notion, a recent study demonstrates that the mammalian FBP phosphatase targets IκB to downregulate inflammation and inhibit colorectal tumorigenesis^177^.

CirB interacts with multiple subunits of the 20S core proteasome, inhibiting its hydrolytic activity. By suppressing proteasome function, CirB prevents the degradation of IκBα, which may lead to prolonged inhibition of the NF‐κB pathway, thereby enhancing bacterial survival within the cell^73^. In addition, a study has suggested that CBU1639 may suppress NFκB‐dependent transcription through either direct or indirect mechanisms, thereby attenuating the pro‐inflammatory response of macrophages to *C. burnetii* infection. However, the precise underlying mechanism remains unclear and warrants further investigation^178^.

## INHIBITION OF RNA SENSING BY RIG‐I

Type I interferons (IFNs) are a group of cytokines that can be induced by various bacterial pathogens to activate cell‐autonomous defense mechanisms. While these cytokines are primarily recognized for their antiviral properties, their role in bacterial infections remains poorly understood^179^. It has been demonstrated that in macrophages infected with *L*. *pneumophila*, the double‐stranded DNA (dsDNA) sensor cGAS and the RIG‐I‐like receptors, which detect dsRNA, are activated. This activation triggers the production of type I IFNs, which helps restrict intracellular bacterial replication^180^, ^181^, ^182^, ^183^, ^184^. In contrast, *C*. *burnetii* infection does not induce the production of type I IFNs^185^, ^186^. However, when THP‐1 macrophages were treated with IFN‐β, the cells became restrictive to *C*. *burnetii* ^81^. A recent study revealed that two T4Es, EmcA and EmcB, inhibit RIG‐I signaling and the production of type I IFNs. Among these, EmcB is a deubiquitinase that targets K63‐linked ubiquitin chains on RIG‐I to block signaling through MAVS^81^. In addition, NopA can inhibit the production of IFN‐α during *C. burnetii* infection by preventing the transcription factor interferon regulatory factor 3 (IRF3) from entering the cell nucleus^70^. Another study found that CBU1314 can block the polymerase‐associated factor 1 (PAF1) complex, which mediates interferon‐stimulated response element‐dependent transcription, thereby inhibiting the production of IFN^81^.

## MODULATION OF HOST METABOLISM

Cholesterol is the major lipid component of eukaryotic cell membranes, influencing both membrane fluidity and permeability. Intracellular cholesterol plays a critical role in various processes, including Golgi trafficking^187^, endocytic transport^188^, and the formation of membrane contact sites between organelles^189^. *C. burnetii* has been shown to exploit cholesterol for cell entry and intracellular replication^190^. During the early stages of CCV formation, an increase in cholesterol concentration leads to excessive acidification of the CCV, resulting in the lytic death of *C. burnetii* ^191^. Researchers have found that adding exogenous cholesterol to cholesterol‐deficient cells leads to the formation of smaller CCVs and reduction in *C*. *burnetii* replication. Furthermore, the number of non‐fused CCVs containing degraded bacteria increased in these cells^191^. Treatment with U18666A, which traps cholesterol in lysosomes and CCVs, results in a decrease in pH^191^, ^192^. Inhibition of vesicular ATPases involved in organelle acidification prevents cholesterol‐induced acidification of CCVs and bacterial death, highlighting the importance of cholesterol homeostasis in CCV development and bacterial replication^191^. At 4 days post‐infection, CCVs significantly expanded, and the expression of genes involved in cholesterol biosynthesis and uptake was transiently upregulated in infected cells. However, by Day 6 post‐infection, the expression of these genes decreased to levels similar to the control group, at which point the expansion of the CCVs has also ceased. This suggests that this upregulation may be in response to the cholesterol demand required for CCV expansion. However, it remains unclear whether this transient upregulation is dependent on the T4SS of *C. burnetii* ^193^. Although *C. burnetii* itself does not detectably synthesize cholesterol, it harbors two homologs of eukaryotic sterol reductases, *cbu1158* and *cbu1206*. The proteins encoded by these genes are likely localized in the bacterial cell membrane and are not considered T4Es^194^. Furthermore, the oxysterol‐binding protein (OSBP)‐related protein 1 long form (ORP1L) is recruited to the CCV membrane, and this recruitment is dependent on the Dot/Icm system^195^. ORP1L is critical for cholesterol‐dependent endosomal transport and the formation of membrane contact sites (MCSs) between late endosomes/lysosomes and the ER^196^. Additionally, it interacts with VAMP‐associated proteins (VAPs) on the cytoplasmic surface of the ER through two phenylalanine residues (FF) in an acidic tract (FFAT) motif^195^. Transmission electron microscopy has revealed tight contacts between the ER and CCV membrane at multiple sites, indicating the presence of MCSs. Knockdown of ORP1L resulted in smaller CCVs, highlighting its critical role in CCV development^195^.

Treatment of *C*. *burnetii*‐infected mouse alveolar macrophages with chloramphenicol has been shown to reduce lipid droplet accumulation in these cells^197^. A similar phenomenon was observed in *C*. *burnetii*‐infected THP‐1 cells, indicating that the decrease in lipid droplet during infection requires protein synthesis in *C*. *burnetii*, probably one or more T4Es^197^. While disruption of the lipid droplet homeostasis clearly affects *C*. *burnetii* growth, the precise mechanism by which *C. burnetii* utilizes fatty acids or sterols released from the breakdown of lipid droplets (LDs) remains unclear. These components may serve as nutrients or be involved in the production of lipid‐derived immune mediators, such as prostaglandin E2^194^. Additionally, CbEPF1 has been shown to colocalize with LDs in host cells and interact with the host VAP proteins through its FFAT motif to establish membrane contact sites between LDs and the ER. This interaction regulates lipid droplet growth in a manner dependent on the FFAT motif^82^.

*C*. *burnetii* is auxotrophic for 11 amino acids^198^, meaning it can only proliferate in environments where these essential nutrients are available^199^, ^200^. The autophagic machinery may be crucial for the transport of these essential amino acids to the CCV, although other pathways might also be involved. Furthermore, the specific roles of individual amino acids in the physiology of *C*. *burnetii*, beyond protein synthesis, and the spatial and temporal regulation of amino acid uptake remain unclear. *C*. *burnetii* encodes several predicted amino acid and peptide transporters, but it is not yet known whether these are essential for intracellular growth. Additionally, it is unclear whether *C*. *burnetii* encodes T4Es that regulate the import of amino acids^201^. *C. burnetii* also utilizes carbohydrates for growth in axenic medium, but it remains unknown whether it employs T4Es to actively manipulate host carbohydrate metabolism^202^.

## CONCLUDING REMARKS

The parasitophorous vacuole created by *C. burnetii* resembles the lysosomal compartment, distinguishing it from other intravacuolar pathogens^203^. As previously mentioned, *C. burnetii* utilizes a cohort of T4Es to modulate endocytic pathways, including the secretion of hydrolytic enzymes such as cathepsin B^124^. This lysosome‐centered pathogenic strategy may serve as a guide for future studies of its T4Es, using such methods as proximity labeling^204^ to identify their cellular targets.

The study of *C. burnetii* pathogenicity has been greatly facilitated by the development of the ACCM medium, which enables genetic manipulation of the bacterium and allows phenotype to be assigned to specific T4E. The observation that inactivation of a single effector gene often leads to defects in virulence suggests that anti‐virulence agents targeting these effectors could be effective in preventing or treating the infection caused by *C. burnetii*. However, several challenges remain in fully understanding the infection mechanism of this important pathogen. First, the precise biochemical activities of most T4Es remain elusive. In many cases, it is unclear whether simple protein‐protein interactions between an effector and its host target are sufficient to cause the observed phenotype. Effectors with distinct biochemical activities represent more promising drug targets than those whose effects are solely mediated through binding. Advances in informatics‐based structure modeling, such as the AlphaFold^205^, will facilitate biochemical studies of these effectors. Another critical question is how *C. burnetii* achieves the temporal regulation during infection. It is evident that distinct sets of effectors are required at specific stages after bacterial uptake. These effectors may be delivered into host cells at the appropriate time through regulatory circuits that govern gene expression in response to cues unique to different infection phases. The strength of the signals recognized by the Dot/Icm apparatus, as well as the stability of the effector itself, also influences its abundance in infected cells after translocation. Third, a better understanding of the coordination among different effectors in supporting the biogenesis and maintenance of the CCV is crucial. A large cohort of *C. burnetii* T4Es has been shown to be required for optimal intracellular replication, and determining which combinations are essential for virulence is of great interest. This goal has become more achievable with the recent development of a base editing method that enables the inactivation of specific genes in *C. burnetii* ^53^, facilitating the identification of the minimal set of T4Es essential for its virulence. Targeting such effectors simultaneously could enhance the efficacy of anti‐virulence‐based therapeutics. Collaborative efforts between microbiologists and scientists with complementary expertise, including structural biologists, biochemists, and medicinal chemists, will accelerate progress in this field, leading to more exciting discoveries in the years to come.
